# Analysis of defensive secretion of a milkweed bug *Lygaeus equestris* by 1D GC-MS and GC×GC-MS: sex differences and host-plant effect

**DOI:** 10.1038/s41598-020-60056-9

**Published:** 2020-02-20

**Authors:** Martina Havlikova, Tereza Bosakova, Georg Petschenka, Radomir Cabala, Alice Exnerova, Zuzana Bosakova

**Affiliations:** 1 Department of Analytical Chemistry, Faculty of Science, Charles University, Prague, Czech Republic; 2 Department of Insect Biotechnology, Justus-Liebig-University Giessen, Giessen, Germany; 3 Toxicology Department, Institute of Forensic Medicine and Toxicology, General University Hospital in Prague, Prague, Czech Republic; 4 Department of Zoology, Faculty of Science, Charles University, Prague, Czech Republic

**Keywords:** Chemical biology, Zoology

## Abstract

The composition of defensive secretion produced by metathoracic scent glands was analysed in males and females of the milkweed bug *Lygaeus equestris* (Heteroptera) using gas chromatography with mass spectrometric detection (GC-MS). The bugs were raised either on cardenolide-containing *Adonis vernalis* or on control sunflower seeds in order to determine whether the possibility to sequester cardenolides from their host plants would affect the composition of defensive scent-gland secretion. Profiles of the composition of defensive secretions of males and females raised on sunflower were closely similar, with predominant presence of (*E*)-2-octenal, (*E*)-2-octen-1-ol, decanal and 3-octen-1-ol acetate. The secretion of bugs raised on *A. vernalis* was more sexually dimorphic, and some chemicals e.g. (*E,E*)-2,4-hexadienyl acetate and 2-phenylethyl acetate were dominant in males, but absent in females. Compared to bugs from sunflower, the scent-gland secretion of bugs raised on *A. vernalis* was characterized by lower overall intensity of the peaks obtained for detected chemicals and by absence of some chemicals that have supposedly antipredatory function ((*E*)-2-hexenal, (*E*)-4-oxo-hex-2-enal, 2,4-octadienal). The results suggest that there might be a trade-off between the sequestration of defensive chemicals from host plants and their synthesis in metathoracic scent-glands.

## Introduction

Chemical defense belongs to the most widespread antipredatory strategies^1,2^ and particularly insects employ a diverse arsenal of chemicals to defend themselves against their antagonists^3–5^. Among insects, the true bugs (Hemiptera: Heteroptera) are characterized by the ancestral and almost universal presence of chemical defenses, that are highly diverse across various true bug taxa and that are frequently associated with aposematic coloration and mimicry^6,7^. Chemical defense of adult true bugs is based on metathoracic scent gland secretions that are released upon attack of a predator^8^. The scent-gland secretion of true bugs is a complex mixture of compounds, some of which could be toxic or irritating to predators while others may act as chemical warning signals perceived either by olfaction (highly volatile compounds) or by taste or chemesthesis^6,9,10^. Very common compounds of defensive secretion of various true bug taxa include short-chain aldehydes, oxo-aldehydes, ketones, alcohols, organic acids, esters, etc^6,11,12^. Nevertheless, the composition of the scent-gland secretion is taxon-specific and sexually dimorphic^13^, and also differs between adults and larvae, which possess a different scent-gland system than the adults (dorsoabdominal scent-glands)^14^. Moreover, the relative content of individual compounds may depend on the season, physiological state and diet^15^.

Sampling of volatile secretions of true bugs is a crucial step of analyses. Majority of published methods require killing the insects (for dissection and extraction of sufficient quantities of secretion)^14,16–19^. Only few studies have been focused on non-lethal sampling, but these procedures usually do not involve any irritation of the insects that would simulate a predator attack and ensure the discharging of defensive secretion^19–21^. In a previous study^11^, we developed a non-lethal sampling method based on irritation of true bugs by pressing them with the plunger of a syringe. This sampling procedure is simple, ensures discharging of defensive secretions as it effectively simulates an attack of a vertebrate predator such as a bird or a lizard, and involves negligible loss of volatile compounds. The method was successfully applied for analysing defensive secretions of three true species of pyrrhocorid bugs by gas chromatography with mass spectrometric detection (GC-MS), combined with a mid-polar column (Rtx-200)^11^.

Defensive secretion of true bugs is a highly complex mixture of diverse compounds demanding high separation efficiency. GC-MS is the most appropriate technique for the identification of volatile compounds. Nevertheless, one-dimensional GC-MS (1D GC-MS) can encounter difficulties with the separation and identification of all the micro- and macro-components during one analysis^22^. Therefore, comprehensive two-dimensional GC equipped with a cryogenic modulation technique and hyphenated with MS (GC×GC-MS) could be more effective. This technique allows sample separation simultaneously on two columns of orthogonal polarities so that almost co-eluting peaks could be separated^23–25^.

In addition to endogenously produced scent-gland defensive secretion, some species of true bugs sequester toxic cardenolides (cardiac glycosides) from their host plants to protect themselves against predators^26–28^. Cardenolides are specific inhibitors of the membrane-bound enzyme Na^+^/K^+^-ATPase, which is essential for many physiological functions in animals. Therefore, cardenolides are highly toxic for a wide range of animal taxa^29^. Nevertheless, several insect taxa have evolved physiological resistance based on strongly reduced binding affinity of their Na^+^/K^+^-ATPase to cardenolides^30,31^. Furthermore, these insects store cardenolides in their bodies (sequestration) and employ them as an antipredatory chemical defense. Sequestration of host-plant cardenolides is characteristic for milkweed bugs (Heteroptera: Lygaeidae: Lygaeinae)^28,32,33^ such as *Oncopeltus fasciatus* that sequesters cardenolides from the seeds of *Asclepias* spp. (Apocynaceae^34^). Sequestered cardenolides are stored in a special system of elaborate subcuticular storage compartments^32,33^, from which they are released upon attack of a predator.

Remarkably, sequestration of cardenolides is a widespread and apparently ancestral trait in the Lygaeinae and occurs also in the Palaearctic species *Lygaeus equestris*^31^. Although *L. equestris* has a specific association with *Vincetoxicum hirundinaria* (Apocynaceae), its host-plant spectrum includes various other plants^35^. Among them, the cardenolide-producing *Adonis vernalis* (Ranunculaceae) is an important host plant for *L. equestris*, and the bugs can sequester cardenolides from this host-plant species (Petschenka *et al*. in prep.). Milkweed bugs, including *L. equestris*, are characterized by their red-and-black aposematic color patterns, and they are protected against both vertebrate^9,36^ and arthropod^37,38^ predators.

As both, the synthesis of defensive chemicals and their sequestration from host plants, are generally regarded as being costly for herbivorous insects^1,39^, it is possible that in those species that use both lines of chemical defense, there might be a trade-off between investments into each of them. Studies directly comparing chemical defenses in polyphagous insects are quite rare, but for example in *Heliconius* butterflies, the ability to synthesize cyanogenic glycosides varies greatly among species and correlates negatively with sequestration of the cyanogens from host plants^40^. Likewise, the results of comparative analysis of costs related to production of autogenous and sequestered defenses in larvae of leaf beetles (Chrysomelidae) indicate greater costs of synthesizing defenses de novo than sequestering them from host plants^5,41^. In a polyphagous leaf beetle *Chrysomela lapponica*, larvae from populations living on salicylic glucoside-rich willows sequester defensive salicylaldehyde, while larvae living on salicylic glucoside-poor host plants autogenously synthesize butyric esters^42^. These results indicate, that in polyphagous species, the individuals feeding on a toxin-containing host plants may thus invest less into the synthesis of autogenous defensive chemicals.

In this study, we analyzed the secretions of metathoracic scent glands in males and females of the milkweed bug *L. equestris* by 1D GC-MS and GC×GC-MS in combination with a commercial cryogenic modulator. A non-lethal method of mechanical irritation of the true bugs and solid phase microextraction (SPME)^11^ were used for secretion sampling. Specifically, we compared the composition of secretion between bugs raised either on a toxic, cardenolide-containing host plant *A. vernalis* or on non-toxic sunflower as a control to test whether there are any intraspecific differences in composition of the secretion connected with feeding on different host plants and between sexes that might indicate a potential trade-off between the defense based on autogenous scent-gland secretion and the defense based on sequestered host-plant cardenolides.

## Materials and Methods

### Insects and plants

Adults of *L. equestris* were originally obtained from an *A. vernalis* habitat north of Lebus, Germany and maintained in the laboratory on hulled sunflower seeds (*Helianthus annuus*; sunflower further on) for multiple generations in an environmental chamber (Binder KBWF 240) at 26–28 °C, 60% relative humidity, and a 16:8 h day/night cycle. Bugs were maintained in gauze-covered plastic containers (19 ×19 ×19  cm) lined with a paper towel and supplied with water from 2 ml Eppendorf tubes plugged with cotton. For the experiment, the eggs of *L. equestris* were transferred to a diet of either pure hulled sunflower seeds or an approximate 1:1 mixture of sunflower and mature seeds of *A. vernalis* (both obtained commercially) ensuring that insects were supplied with both types of seeds *ad libitum*. Previous experiments revealed that *L. equestris* always access *A. vernalis* seeds and sequester cardenolides when presented in seed mixtures (Petschenka *et al*. in preparation). Insects were maintained under the same conditions as described above in an environmental chamber (Fitotron SGC 120, Weiss Technik, Loughborough, UK) for roughly three weeks. After reaching the adult stage, the bugs were subjected to chemical analysis.

### Defensive compounds extraction

A non-lethal mechanical irritation method^11^ was used for sampling the defensive secretion. The bugs were stressed by pressing them with the syringe plunger, which regularly resulted in release of the scent-gland secretion. One bug individual was placed in a syringe with a barrel volume of 12.5 mL (Eppendorf, Hamburg, Germany), and the syringe was tempered (at 40 °C for 1 minute) in an incubating shaker (bioSan, Riga, Lithuania). Then the bug was carefully compressed with the plunger of the syringe until the bug could not move anymore. The syringe tip was then closed with a rubber stopper. The compression lasted until droplets of liquid appeared ventro-laterally at the metathorax of the bug. Subsequently, the rubber stopper was removed from the syringe tip, 5 mL of air was drawn in and a SPME fiber coated with triple phase 50/30 μm divinylbenzene/carboxen/polydimethylsiloxane (DVB/CAR/PDMS) located in a manual SPME holder (Supelco, Bellefonte, PA, USA) was immediately inserted into the tip of the barrel. The syringe with the bug and the SPME fiber was placed in the temperature-controlled shaker again. The extraction procedure was based on our previous study^11^ and the adsorption time (30–120 min) and temperature (25–45 °C) were optimized with incubation for 90 min at 40 °C.

The SPME fiber was thoroughly cleaned and conditioned for 15 min before and after each analysis in an external syringe oven at 250 °C under vacuum. Before each analysis of a defensive secretion, we carried out control analysis of the cleaned SPME fiber (without sorption of analytes) and then with sorption in the clean vacant syringe to identify background compounds. The analyses of secretions obtained from individual bugs within each of the four groups (males and females raised either on *A. vernalis* or sunflower) were carried out in triplicate (*n* = 3), i.e. three milkweed bugs were used in total per group and each one was used for secretion collection three times (but not each collection provided release of scent-gland secretion). In parallel, a measurement using a non-irritated bug that underwent the same procedure as for secretion sampling except for being pressed with the plunger was performed as a blank.

### Chemical analysis

The 1D GC-MS analysis was performed using a gas chromatograph with a quadrupole mass spectrometer GCMS-QP2010 Ultra instrument (Shimadzu, Kyoto, Japan). The instrument was equipped with a mid-polar Rtx-200 column - 20 m × 0.15 mm i.d., 0.15 μm film thickness (trifluoropropylmethyl polysiloxane stationary phase, Restek, USA) or with a low-polar SLB-5ms column - 30 m × 0.25 mm i.d., 0.25 μm film thickness (5% diphenyl/95% polydimethylsiloxane stationary phase, Supelco, USA). Helium (99.999%, Linde, Czech Republic) was used as a carrier gas at a constant linear flow rate of 35 cm s^−1^. Splitless-mode injection (1 min) with SPME liner at 250 °C was employed for analysis of the true bugs’ secretion. For measurement of the standard solutions, 1 μl of the sample was injected by an auto-sampler (AOC-20i, Shimadzu) in the split mode (split ratio, 1:50).

The oven temperature gradient was set as follows: 35 °C was held for 3 min, ramped at 5 °C min^−1^ to 130 °C, then ramped at 20 °C min^−1^ to 300 °C and maintained for 5 min (the total run time was 35.5 min).

The mass spectrometer was operated in the full scan mode (*m*/*z* 35–500). The ion source and interface temperatures were 200 and 250 °C, respectively.

A Shimadzu gas chromatograph with quadrupole mass spectrometer GCMS-QP2010 Plus (Shimadzu, Kyoto, Japan) equipped by a cryogenic modulator Zoex ZX1 (Zoex Corporation, Houston, USA) with a two-stage loop liquid nitrogen modulator was used for the GC×GC-MS analysis. The low-polar column SLB-5ms - 30 m × 0.25 mm i.d., 0.25 μm film thickness (5% diphenyl/95% polydimethylsiloxane stationary phase, Supelco, USA) was used for the first dimension and polar Supelcowax 10 - 1 m × 0.1 mm i.d., 0.1 μm film thickness (poly(ethylene glycol) stationary phase, Supelco, USA) for the second dimension. Another 2 m of the second dimension column was used as a loop.

The cryogenic modulation conditions were: 400 kPa inlet column pressure, modulation period 4 s, duration of hot pulse 375 ms and temperature of hot jet 350 °C. All other parameters were the same as for the 1D GC-MS measurement.

1D GC and GC×GC data were collected and evaluated by GCMS solution software version 2.7 (Shimadzu, Kyoto, Japan) and transformation and visualization of GC×GC data were carried out by GC Image software (version 2.0, Zoex Corporation, Houston, USA). Secretion components were identified by comparing the obtained spectra with those in the NIST 2008 Mass Spectra Library, and the identification was confirmed by comparing the retention times and mass spectra with those obtained from measurements of reference compounds. Identification by library reference only was used for substances for which a standard was not available, but only if the match factor in the NIST 2008 database was higher than 85%. OriginPro 2015 (Origin Lab Corporation, Northampton, MA, USA) software was used for data processing.

### Chemicals

A mixture of *n*-alkanes (C6–C10) dissolved in pentane was purchased from Fluka (Buchs, Switzerland) and α-pinene (≥95%) from Merck. All other reference compounds were supplied by Sigma-Aldrich (Munich, Germany): (*E*)-2-hexen-1-ol (≥ 95%), (*E*)-2-octenal (≥94%), (*E*)-2-nonenal (≥93%), hexyl acetate (99%), 1-decyne (98%), (*Z*)-3-hexenyl acetate (≥98%), (*E*)-2-decenal (analytical standard), 3-methyl-1-butanol acetate (isoamyl acetate) (≥97%), benzaldehyde (≥99%), nonanal (97%), (*E*)-2-hexenal (98%), (*E*)-2-octen-1-ol (97%), decanal (≥98%), 2,4-octadienal (96%), (*E,E*)-2,4-decadienal (analytical standard), (*R*)-(+)-limonene (97%), (*E,E*)-2,4-hexadienal (≥95%). Dichloromethane (anhydrous, ≥99.8%) and pentane (anhydrous, ≥99%) used as diluents were also from Sigma-Aldrich.

Samples of the reference compounds were prepared by dilution of the individual compounds in dichloromethane, except for a mixture of alkanes which were diluted in pentane. The concentration of stock solutions of each compound was 0.1 mg mL^−1^ but the final dilution of each standard was individually modified to obtain good reproducibility of retention times.

### Statistical analysis

For comparison of similarity among the four studied groups of bugs, we used principal component analysis (PCA). For qualitative comparison, the data matrix was based on presence or absence of individual compounds in the defensive secretion, obtained by the analyses conducted in triplicate, as described in Tab. 1. PCA was carried out using Statistica software (version 8.0, StatSoft Inc., Tulsa, OK, USA).

## Results and Discussion

### One-dimensional GC-MS analysis of the *L. equestris*’ defensive secretion

**Figure 1:**
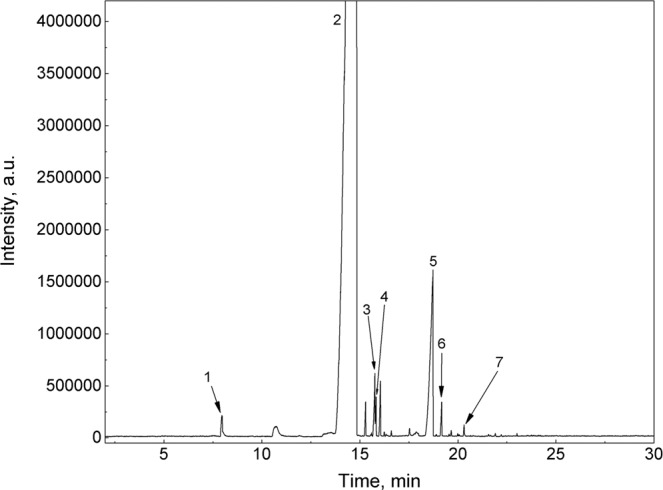
Chromatogram obtained by SPME-GC-MS analysis of the defensive secretion of *L. equestris* males raised on sunflower seeds on mid-polar Rtx-200 column; identified analytes: (1) 3-hexenal, (2) coeluting decanal, (*E*)-2-octenal and nonanal, (3) 2,4-octadienal, (4) 3-octen-1-ol acetate, (5) cyclooctanol, (6) (*E*)-2-decenal, (7) undec-2-enyl acetate.

For the pilot sampling of the defensive secretion of *L. equestris* males raised on sunflower seeds and its subsequent GC analysis, we employed the methodology described by Krajíček *et al*.^11^. As the sampling method by SPME fibre yielded peaks with very high response intensity, only one true bug individual was used for collection of the secretion, instead of three, as described by Krajíček *et al*.^11^. The sample was separated on a medium polar Rtx-200 column, which is considered to be universal. As can be seen in Fig. 1, individual components of the defensive secretion were not adequately separated on this column, compared to the defensive secretions of the true bug species analysed in a previous study^11^ and the spectrum had substantially fewer peaks. This fact was most obvious in the area around 13–15 min, where there was only one peak in the chromatogram. According to the data obtained in separation of the individual standards on the Rtx-200 column under the same conditions^11^ it can be assumed that substances, such as nonanal, (*E*)-2-octenal, or decanal, co-eluted in this area. Nevertheless, several other analytes were identified using this column (see Fig. 1).

**Figure 2:**
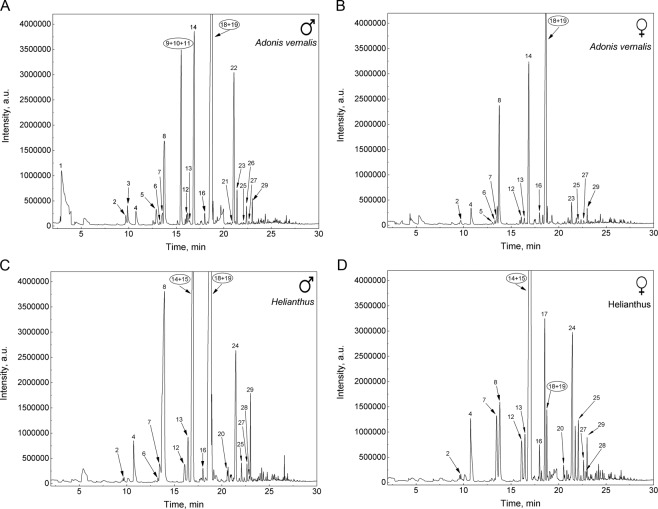
SPME-GC-MS analysis of the defensive secretion of *L. equestris*. Comparison of males and females raised on different host plants: toxic *A. vernalis* and non-toxic sunflower used as a control. Numbering of the peaks corresponds to Table 1.

Consequently, in the next experiment, a substantially less polar SLB-5ms column was employed, on which much better separation was obtained under the same experimental conditions, and thus all the subsequent 1D GC-MS analyses were performed on this column. The representative chromatograms obtained for males and females from both host plants are depicted in Fig. 2. Only peaks with a response greater than 20,000 a.u. (arbitrary units) and whose occurrence was confirmed by analysis of scent-gland secretions released by three individuals (one at a time) were chosen for identification.

**Table 1:** The compositions of the defensive secretion released by males and females of *L. equestris* raised either on toxic *A.vernalis* or sunflower (*Helianthus annuus*) as a non-toxic control obtained by 1D GC-MS conducted in triplicate; peak numbers (same as in Fig. 2), means of retention times with standard deviation (*t*_ret_), means of relative peak areas expressed as percentages with standard deviation, plus symbol (+) denotes presence of the compound (number of plus symbols indicates relative peak area value - approximately one plus symbol equals relative peak area up to 5%, two plus symbols up to 20% and three plus symbols over 20%), and the identification method.

Peak number	*t*_ret_ (min)	Compound	Male_*Helianthus*_		Female_*Helianthus*_		Male_*A.vernalis*_		Female_*A.vernalis*_		Identification
1	3.06 ± 0.03	1-Methyl-1,3-cyclopentadiene	−	−	−	−	7.34 ± 1.25	++	−	−	B
2	9.69 ± 0.02	3-Methyl-1-butanol acetate	0.08 ± 0.01	+	0.17 ± 0.05	+	0.55 ± 0.16	+	0.50 ± 0.06	+	A, B
3	9.87 ± 0.03	2,4-Hexadien-1-ol	−	−	−	−	1.13 ± 0.17	+	−	−	B
4	10.72 ± 0.04	(*E,E*)-2,4-Hexadienal	2.27 ± 0.48	+	4.52 ± 0.28	+	1.19 ± 0.11	+	2.61 ± 0.10	+	A, B
5	12.88 ± 0.02	2-Ethyl furan	−	−	−	−	1.04 ± 0.03	+	0.28 ± 0.07	+	B
6	13.27 ± 0.06	(*Z*)-3-Hexenyl acetate	0.14 ± 0.04	+	−	−	0.14 ± 0.05	+	0.31 ± 0.09	+	A, B
7	13.46 ± 0.04	3-Methyl-4-methylene-hexane	1.23 ± 0.24	+	5.13 ± 0.71	++	0.84 ± 0.20	+	0.52 ± 0.20	+	B
8	13.88 ± 0.09	Cyclooctanol	14.02 ± 1.02	++	5.31 ± 0.26	++	7.08 ± 0.52	++	15.82 ± 1.25	++	B
9*	**15.53 ± 0.05**	**(*E,E*)-2,4-Hexadienyl acetate**	−	−	−	−	**12.18 ± 1.58**	++	−	−	B
10*		**2,6-Dimethyl-7-octen-2-ol**	−	−	−	−			−	−	B
11*		**1-Octanol**	−	−	−	−			−	−	B
12	16.09 ± 0.02	1-Ethyl-1-methyl-cyclopentane	0.72 ± 0.18	+	2.67 ± 0.32	+	0.37 ± 0.01	+	0.59 ± 0.10	+	B
13	16.46 ± 0.05	5-Ethyl-2(5H)-furanone	2.19 ± 0.55	+	2.72 ± 0.20	+	0.22 ± 0.02	+	0.49 ± 0.11	+	B
14*	**16.90 ± 0.07**	**(*E*)-2-Octenal**	**21.53 ± 2.6**	+++	**50.33 ± 3.12**	+++	13.41 ± 1.56	++	19.11 ± 1.47	++	A, B
15*		**(*E*)-2-Octen-1-ol**					−		−	−	A, B
16	17.99 ± 0.02	2,4-Octadienal	0.51 ± 0.13	+	1.21 ± 0.06	+	0.41 ± 0.04	+	0.99 ± 0.08	+	A, B
17	18.54 ± 0.04	4-Ethylcyclohexanol	−	−	5.84 ± 0.70	++	−	−	−	−	B
18*	**18.70 ± 0.07**	**3-Octen-1-ol acetate**	**44.45 ± 3.49**	+++	**4.15 ± 0.74**	+	**37.93 ± 3.63**	+++	**43.23 ± 1.77**	+++	B
19*		**Decanal**									A, B
20	20.55 ± 0.04	2-Propyl-cyclohexanone	0.23 ± 0.03	+	0.48 ± 0.02	+	−		−		B
21	20.89 ± 0.05	1-Ethyl-cyclopentanol	−	−	−	−	0.07 ± 0.01	+	−	−	B
22	21.07 ± 0.02	2-Phenylethyl acetate	−	−	−	−	9.88 ± 1.10	++	−	−	B
23	21.37 ± 0.06	3-Methyl-4-undecene	−	−	−	−	1.07 ± 0.22	+	1.36 ± 0.15	+	B
24	21.46 ± 0.04	2-Ethyl-cyclohexanone	5.28 ± 0.14	++	7.66 ± 1.44	++	−	−	−	−	B
25	22.07 ± 0.05	(*E*)-2-Decenal	0.40 ± 0.05	+	1.69 ± 0.40	+	0.05 ± 0.02	+	0.20 ± 0.03	+	A, B
26	22.39 ± 0.02	Butanoic acid, undec-2-enyl ester	−	−	−	−	0.51 ± 0.07	+	−	−	B
27	22.64 ± 0.05	4,5-Dimethyl-2-cyclohexen-1-one	0.39 ± 0.02	+	0.56 ± 0.02	+	0.08 ± 0.02	+	0.22 ± 0.08	+	B
28	22.87 ± 0.03	(*E,E*)-2,4-Decadienal	0.11 ± 0.03	+	0.36 ± 0.07	+	−	−	−	−	A, B
29	22.99 ± 0.04	(*E*)-2-Decenyl acetate	1.93 ± 0.04	+	1.03 ± 0.12	+	0.59 ± 0.10	+	0.75 ± 0.11	+	B

*Bold – co-elution in the 1D GC-MS.

The methods used for the identification: A - retention time and mass spectrum of the relevant substance was compared with the reference compound; B - the mass spectrum of the relevant substance was compared with NIST 2008 MassSpectraLibrary.

In order to eliminate misleading results, the peak numbering in Fig. 2 corresponds to all identified components in the defensive secretions, although some compounds co-eluted (large peaks marked with numbers in a circle). These components could not be separated in the 1D GC-MS system and the identification was performed later by GC×GC-MS. The composition of the defensive secretion together with the retention times of the compounds are summarised in Tab. 1. In order to estimate the representation of individual substances in the secretion, the means of relative peak areas (*n* = 3), obtained by a method of the internal normalization and expressed as percentages, are also included in Tab. 1. The percentages of each component represent its relative abundance in the chromatogram. The means of relative peak areas are also expressed by the plus symbols (in parentheses); the higher the number of plus symbols, the higher the relative peak area value (approximately, one plus symbol equals the relative peak area up to 5%, two plus symbols up to 20% and three plus symbols over 20%). This simplification is also advantageous for comparison with GC×GC-MS results.

### Two-dimensional GC×GC-MS analysis of *L. equestris*’ defensive secretion

**Figure 3:**
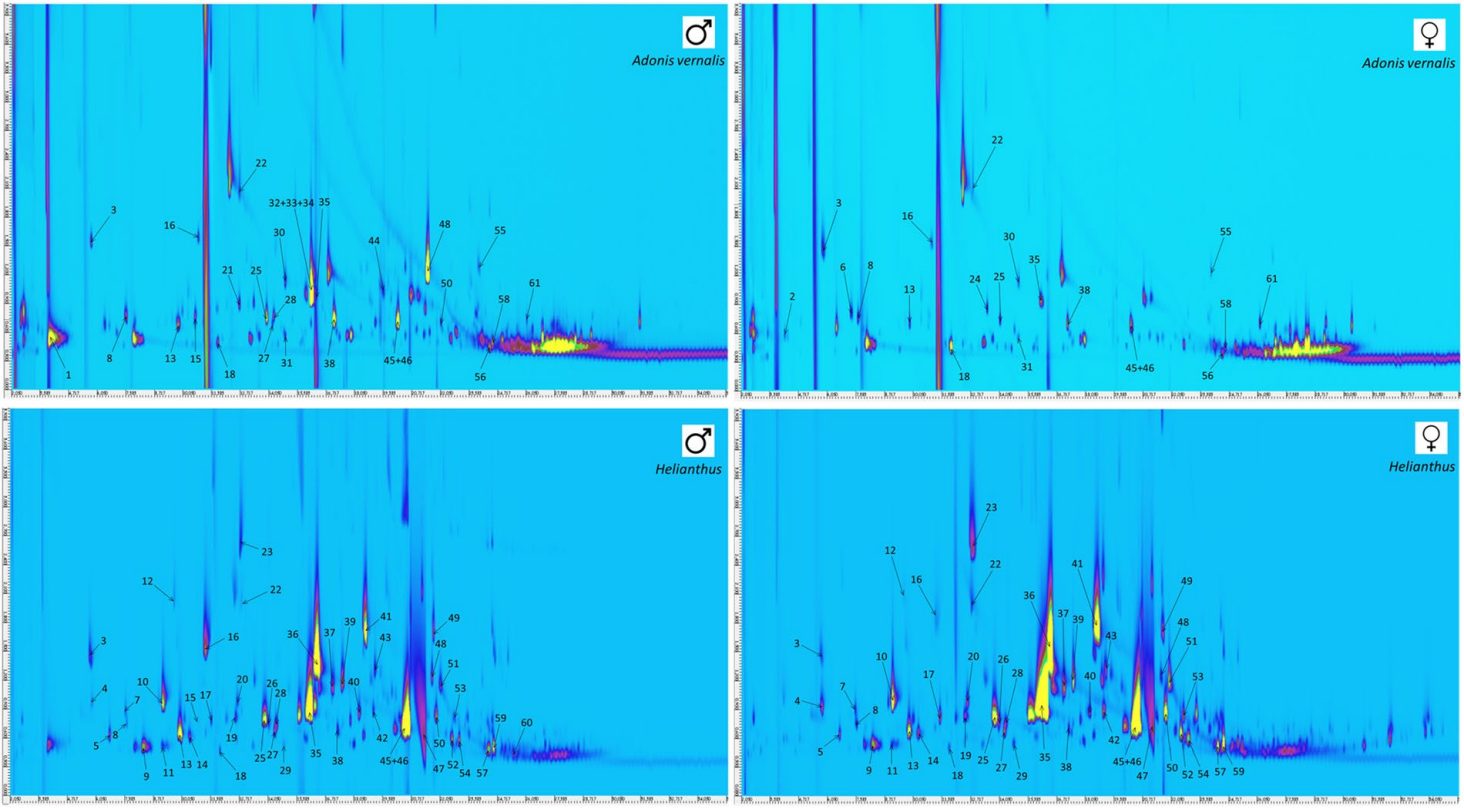
SPME-GC×GC-MS analysis of the defensive secretion of *L. equestris*. Comparison of males and females raised either on toxic *A. vernalis* or the control, non-toxic sunflower seeds. Numbering of the peaks corresponds to Table 2.

Because of co-elution of some components of the defensive secretion in the 1D GC-MS system, analysis using GC×GC-MS in an orthogonal system of a low-polarity SLB-5ms column in the first dimension and a polar Supelcowax 10 column in the second dimension under the conditions described above was performed with the same sampling method as for 1D GC-MS. Secretions from males and females of *L. equestris* raised on toxic *A. vernalis* or sunflower as a control were measured, and the resultant contour plots are depicted in Fig. 3. The composition of defensive secretions was identified by comparing the obtained spectra with the Mass Spectra Library and obtained retention times with standard retention times, if available, as described in the section Material and Methods. The identified components are listed in Tab. 2 and only compounds whose occurrence was confirmed by analysis of scent-gland secretions released by three individuals (one at a time) were chosen for identification. The software used for the evaluation of blobs in GC×GC-MS does not allow for the application of the internal normalization method (as in 1D GC-MS) The plus symbol indicates only the presence of a given compound in the studied defensive secretions, and their number gives a visual estimate of the intensity of the individual blob (the yellow colour represent the highest intensity, see Fig. 3). This facilitates comparison of the results obtained in the different groups tested.

**Table 2:** The compositions of the defensive secretion released by males and females of *L. equestris* raised either on toxic *A. vernalis* or sunflower (*Helianthus annuus*) as a non-toxic control obtained by GC×GC-MS conducted in triplicate; peak numbers (same as in Fig. 3), retention times in the first (*t*_ret_1_) and second (*t*_ret_2_) dimension, +/− symbols denote presence or absence of a particular compound (number of plus symbols indicates intensity of the individual blob) and the identification method.

Peak number	*t*_ret_1_ (min)	*t*_ret_2_ (min)	Compound	Male_*Helianthus*_	Female_*Helianthus*_	Male_*A.vernalis*_	Female_*A.vernalis*_	Identification
1	3.78	0.51	1-Methyl-1,3-cyclopentadiene	−	−	++	−	B
2	4.05	0.54	3-Methyl-butanal	−	−	−	+	B
3	5.78	1.47	3-Methyl-1-butanol	+	+	+	+	B
4	5.85	0.93	2-Methyl-2-butenal	+	+	−	−	B
5	6.58	0.63	Isobutyl acetate	+	+	−	−	B
6	7.12	0.81	2-Hexanone	−	−	−	+	B
7	7.32	0.84	3-Hexenal	+	+	−	−	B
8	7.45	0.72	Hexanal	+	+	+	+	B
9	8.25	0.51	1,3-Octadiene	+	+	−	−	B
10	9.05	0.96	(*E*)-2-Hexenal	++	++	−	−	A, B
11	9.05	0.51	2,4-Octadiene	+	+	−	−	B
12	9.58	2.04	(*E*)-2-Hexen-1-ol	+	+	−	−	A, B
13	9.92	0.63	3-Methyl-1-butanol acetate	++	++	+	+	A, B
14	10.30	0.59	2-*n*-Butyl-furan	+	+	−	−	B
15	10.61	0.75	Heptanal	+	−	+	−	B
16	11.05	1.53	(*E,E*)-2,4-Hexadienal	++	+	+	+	A, B
17	11.25	0.81	2-Methylbut-2-en-1-yl acetate	+	+	−	−	B
18	11.78	0.45	α-Pinene	+	+	+	++	A, B
19	12.52	0.81	3-Ethyl-phenol	+	+	−	−	B
20	12.58	0.96	(*E*)-2-Heptenal	+	+	−	−	B
21	12.72	0.87	2-Ethyl furan	−	−	+	−	B
22	12.72	2.01	Benzaldehyd	+	+	+	+	A, B
23	12.72	2.61	(*E*)-4-Oxo-2-hexenal	+	++	−	−	B
24	13.45	0.84	6-Methyl-5-hepten-2-one	−	−	−	+	B
25	13.85	0.75	Cyclooctanol	++	++	++	+	B
26	14.04	0.75	Octanal	+	+	−	−	B
27	14.32	0.66	Hexyl acetate	+	+	+	−	A, B
28	14.38	0.72	2-Hexen-1-ol acetate	+	+	+	−	B
29	14.72	0.63	5-Decyne	+	+	−	−	B
30	14.78	1.11	2-Ethyl-1-hexanol	−	−	+	+	B
31	14.78	0.51	(*R*)-(+)-Limonene	−	−	+	+	A, B
32	15.98	1.20	(*E,E*)-2,4-Hexadienyl acetate	−	−	+++	−	B
33	16.11	1.14	2,6-Dimethyl-7-octen-2-ol	−	−	++	−	B
34	16.12	0.90	1-Octanol	−	−	++	−	B
35	15.90	0.90	(*E*)-2-Octenal	+++	+++	++	++	A, B
36	16.20	1.35	(*E*)-2-Octen-1-ol	+++	+++	−	−	A, B
37	16.98	1.11	3-Propyl-cyclohexene	+	+	−	−	B
38	17.25	0.66	Nonanal	+	+	++	++	A, B
39	17.45	1.17	2,4-Octadienal	++	++	−	−	A, B
40	18.25	0.84	3-Nonen-2-one	+	+	−	−	B
41	18.58	1.68	2-Butyl-cyclohexanone	++	+++	−	−	B
42	18.91	0.84	(*E*)-2-Nonenal	+	+	−	−	A, B
43	18.98	1.00	2-Pentyl-cyclohexanone	+	+	−	−	B
44	19.38	1.29	(+)-Menthol	−	−	+	−	B
45	20.30	0.65	Decanal	+++	+++	++	++	A, B
46	20.30	0.91	3-Octen-1-ol acetate	+++	+++	+	++	B
47	21.12	0.63	2-Octenoic acid	++	++	−	−	B
48	21.65	1.23	2-Phenylethyl acetate	+	+	+++	−	B
49	21.72	1.68	5-Butyldihydro-2(3 H)-furanone	+	+	−	−	B
50	21.78	0.78	(*E*)-2-Decenal	+	++	+	−	A, B
51	22.05	1.11	2-Decen-1-ol	+	+	−	−	B
52	22.45	0.63	cis-Non-3-enyl acetate	+	+	−	−	B
53	22.65	0.84	(*E,E*)-2,4-Decadienal	+	+	−	−	A, B
54	22.92	0.57	Undec-2-enyl acetate	+	+	−	−	B
55	23.70	1.26	*n*-Decanoic acid	−	−	+	+	B
56	24.32	0.39	Tetradecane	−	−	+	+	B
57	24.32	0.45	Butanoic acid, 1-ethenylhexyl ester	+	+	−	−	B
58	24.45	0.45	Dodecanal	−	−	+	+	B
59	24.52	0.48	(*E*)-2-Decenyl acetate	+	+	−	−	B
60	25.51	0.42	1-Dodecen-3-ol	+	−	−	−	B
61	26.05	0.72	*n*-Dodecanoic acid	−	−	+	+	B

The methods used for the identification: A - retention time and mass spectrum of the relevant substance was compared with the reference compound; B - the mass spectrum of the relevant substance was compared with NIST 2008 MassSpectraLibrary.

**Figure 4:**
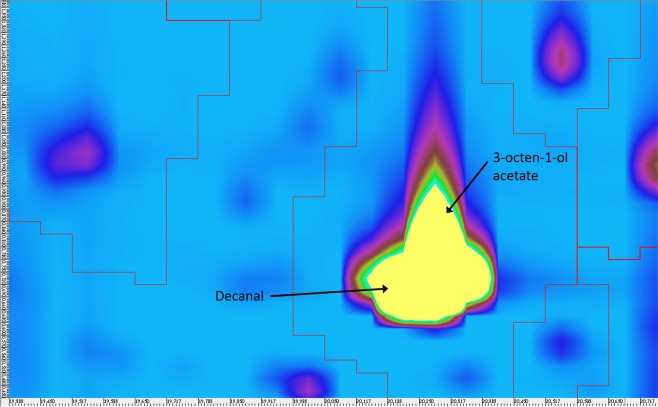
Cut-out of the contour plot obtained for the defensive secretion of the males of *L. equestris* raised on sunflower seeds. Identification of co-eluted compounds.

The use of the SLB-5ms column both in 1D GC-MS and in the first dimension of GC×GC-MS enabled subsequent comparison of the obtained data. The use of GC×GC-MS facilitated the identification of a larger number of analytes compared to the use of 1D GC-MS and, in particular, it was possible to identify the individual components of the peaks, which co-eluted in the 1D system, exhibited the highest intensities and probably represented the most important components of the defensive secretion. This corresponded to the peak with a retention time of 16.9 min in the 1D-GC-MS system, which could be separated into two peaks using the GC×GC-MS system that were identified as (*E*)-2-octenal and (*E*)-2-octen-1-ol at times 15.90 and 16.20 min, respectively. Unfortunately, the peak eluting at time 18.7 min (in 1D GC-MS) could not be completely separated into its individual components even using GC×GC-MS, but they could be at least identified. In this case, the substances decanal and 3-octen-1-ol acetate were identified (both at time 20.30 min in Fig. 4). The four analytes mentioned above were the substances with the greatest relative contents in the defensive secretion and they are likely to play an important role in either antipredatory defence or intraspecific communication of Heteroptera. Especially aldehydes, e.g. C_6_ and C_8_ (*E*)-2-alkenals and (*E*)-4-oxo-2-alkenals, are substances that are important for defence against predators in true bugs^43^.

For the peak eluting in the 1D GC-MS system at time 15.5 min, the separation in the GC×GC-MS system was not sufficiently effective; however, it allowed for identification of the individual components as 1-octanol, 2,6-dimethyl-7-octen-2-ol and (*E,E*)-2,4-hexadienyl acetate (see the differences between sexes). On the other hand, at retention times of 12.72 and 14.78 min (for GC×GC-MS), it was possible to completely separate the pair benzaldehyde and (*E*)-4-oxo-2-hexenal and the pair (*R*)-(+)-limonene and 2-ethyl-1-hexanol.

The intensities in responses of individual components of the defensive secretion can be compared in the contour plot obtained using GC×GC-MS. The contour plots depicted in Fig. 3 were obtained under the same conditions and equally adjusted by the evaluating GC Image software. It is thus possible to observe marked differences in the intensities of the responses of the individual peaks between males and females and especially between individuals raised on different host plants.

Because of the usage of the same sampling method, we could directly compare our results with those reported by Krajíček *et al*.^11^ for three true bug species from the family Pyrrhocoridae. Analytes belonging to the most abundant and thus probably representing the most important components of the defensive secretion of *L. equestris* were also identified in at least one of the three pyrrhocorid species analysed by Krajíček *et al*.^11^. These analytes were (*E*)-2-octenal, decanal and (*E*)-2-octen-1-ol. Thirteen additional substances that occurred in the secretion of sunflower-raised *L. equestris* matched the compounds found in at least one of the three studied pyrrhocorid species including (*E,E*)-2,4-hexadienal, 3-methyl-1-butanol acetate, 2-ethyl-cyclohexanone, 2-hexen-1-ol acetate, and undec-2-enyl acetate^11^.

The composition of the metathoracic scent-gland secretion was analysed in several other species of milkweed bugs (Lygaeinae), though using different methods of secretion sampling and analysis, and therefore the results are not entirely comparable. Nevertheless, short-chained aldehydes (namely (*E*)-2-hexenal and (*E*)-2-octenal), alcohols and acetates were dominant also in secretions of metathoracic scent-glands in *Lygaeus kalmii*, *Oncopeltus fasciatus*, *O. cingulifer*, *O. unifasciatellus*, *Tropidothorax cruciger*, and *Spilostethus rivularis*^43–45^.

### Differences in the composition of defensive secretion between males and females

There were minimal differences between males and females of *L. equestris* raised on sunflower seeds. With regard to the diversity of substances, the compositions were practically identical in both sexes. Differences were found especially in ratios of the intensities of the largest peaks. In males, the predominant components of the secretion were decanal and 3-octen-1-ol acetate and, compared to the females, the peak of cyclooctanol was more intense, while in females, the predominant peaks were those corresponding to (*E*)-2-octen-1-ol and (*E*)-2-octenal. Most of the simple alcohols belong particularly to the group of insect attractants and, in various ratios, especially the above two alcohols indicate that these substances also have a pheromonal function^46^. In addition, across all measurements, about a one third larger amount of branched cyclohexanones was identified in females (these were allyl-cyclohexanones and, based on the spectral library, most probably 2-pentyl-cyclohexanone and 2-butyl-cyclohexanone). These substances are usually considered to be pheromones^47^.

**Figure 5:**
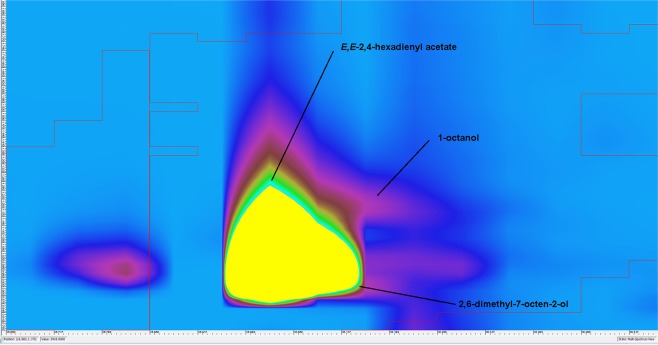
Cut-out of the contour plot obtained for the defensive secretion of *L. equestris* males raised on *A. vernalis* seeds. Identification of co-eluted compounds.

Differences between females and males raised on seeds of *A. vernalis* were found not only in the intensity of the response of individual components, but we also found qualitative differences. In females, the intensity of obtained peaks was substantially lower than in males. This phenomenon could be associated with a higher amount of cardenolides sequestered from host plants by the females as described for the monarch butterfly (*Danaus plexippus*)^48^. The greatest difference in the composition of the defensive secretion is represented by the peak at a retention time of 15.5 min (for 1D GC-MS) found only in males (almost the largest in GC×GC-MS). Identification of the components of this peak was impossible when using 1D GC-MS and adequate separation was not achieved even with GC×GC-MS (see Fig. 5). Most probably, three substances co-eluted here and, on the basis of point spectra at the three most intense points in the blob, three analytes were identified as 1-octanol, 2,6-dimethyl-7-octen-2-ol and (*E,E*)-2,4-hexadienyl acetate and the last mentioned compound was the most abundant.

Another important difference between males and females raised on *A. vernalis* was the absence of 2-phenylethyl acetate in the females’ chromatograms. This compound was the fourth largest peak in the 1D GC-MS chromatograms from males, and in the GC×GC-MS contour plots it was amongst the four most intense peaks. Both these acetates, found only in males, were also found only in secretion of males of other milkweed bugs (e.g. *Neacoryphus bicrucis*) as pheromones attracting both sexes^44,45,49^. The presence of these acetates in males raised on *A. vernalis* also supports the hypothesis that toxins sequestered from host plants suppress the defensive role of the metathoracic scent glands in milkweed bugs, and therefore the glands can serve their other function in producing pheromones^6,50^.

**Figure 6:**
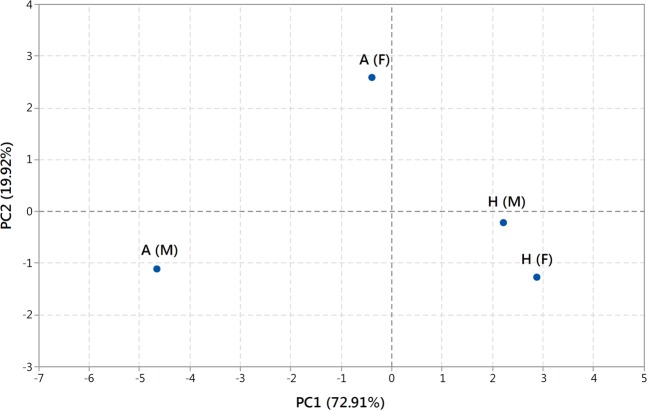
PCA of the composition of defensive secretions in males and females of *L. equestris* raised on different host plants. The first principal component (PC1) explains 72.91% of variance and the second principal component (PC2) explains 19.92% of variance. Symbols: H(M) males and H(F) females raised on sunflower seeds, A(M) males and A(F) females raised on *A. vernalis* seeds.

The data based on presence or absence of individual compounds in defensive secretions of males and females from different host plants were analysed means of PCA (Fig. 6). The first two principal components (PC1 and PC2) together explained for 92.83% of total variance. Males and females raised on sunflower seeds are closely similar, whereas males and females raised on *A. vernalis* considerably differ from each other.

### Effect of host-plant toxicity on composition of defensive secretion

**Figure 7:**
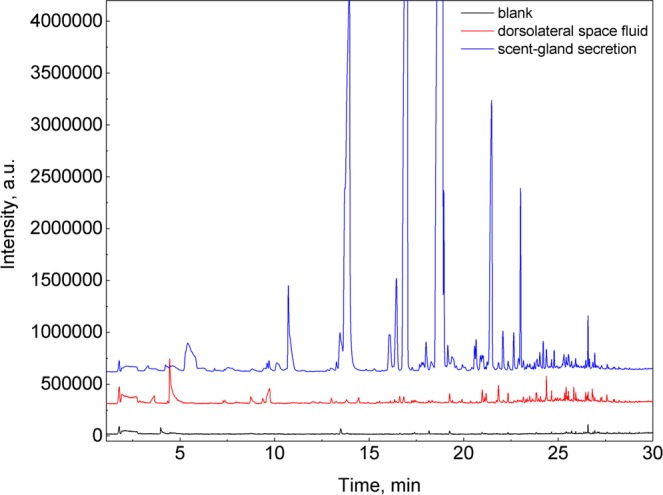
Comparison of the blank, dorsolateral space fluid and scent-gland secretion. SPME-GC-MS analysis of the scent-gland secretion of the male *L. equestris* raised on sunflower seeds and the blank and the dorsolateral space fluid released by male of *L. equestris* raised on *A. vernalis*. Sampling of the secretion was made by compression with the plunger of a syringe, for obtaining the blank this step was omitted.

When pressed with the plunger during the secretion sampling, we observed that the bugs raised on *A. vernalis* showed a substantially lower willingness to release the scent-gland secretion. Instead, they frequently released defensive fluid from the dorsolateral space (probably containing cardenolides^32,51^). This fluid was also sampled using SPME fibres in the same way as the scent-gland secretion. When the chromatogram of the dorsolateral space fluid was compared with a blank, (see Fig. 7) only very few volatile components could be detected. Based on GC×GC-MS chromatograms we only identified a small amount of octanal, nonanal, decanal and also α-pinene. Releasing the dorsolateral space fluid as a response to pressure corresponds with the presence of an elaborate system of subcuticular storage compartments^32^, which allows the quick discharge of the fluid upon predator attack.

More importantly, scent-gland secretion of the bugs raised on *A. vernalis* contained a smaller number of chemicals compared to the secretion of sunflower-raised bugs. Moreover, for most of the chemicals that were present in secretions of bugs from both host plants, the overall intensity of the peaks obtained was lower in the bugs raised on *A. vernalis* than in the bugs from sunflower (Tab. 2, Fig. 3). Out of 14 chemicals that were dominant in the secretion of sunflower-raised bugs of both sexes (Tab. 2), only 2 (cyclooctanol and α-pinene) were equally dominant in the secretion of bugs raised on *A. vernalis*. Six others were present in a much lower amount (e.g. (*E*)-2-octenal, decanal, 3-octen-1-ol acetate) and 6 were entirely absent (e.g. (*E*)-2-hexenal, (*E*)-4-oxo-2-hexenal, (*E*)-2-octen-1-ol). Contrastingly, out of the chemicals dominant in the secretion of the bugs raised on *A. vernalis*, only 4 (e.g. 2-phenylethyl acetate, (*E,E*)-2,4-hexadienyl acetate) were absent in secretion of sunflower-raised bugs, all of them being sex specific and present only in males (see above). In general, the secretion of individuals raised on *A. vernalis* contained mostly esters, variously branched alcohols, and some aldehydes and, amongst substances eluting later, also higher carboxylic acids, whereas the secretion of bugs raised on sunflower was dominated by aldehydes and oxo-aldehydes and also some alcohols and esters.

The absence (or low amount) of some of the aldehydes (3-hexenal, (*E*)-2-hexenal, (*E*)-2-octenal, heptanal, octanal, 2,4-octadienal, (*E*)-2-nonenal, (*E,E*)-2,4-decadienal) and oxo-aldehydes ((*E*)-4-oxo-2-hexenal) in the secretion of bugs raised on *A. vernalis* is striking, as they are considered to be important constituents of true-bug antipredatory defence, being aversive^52^ or toxic for arthropod predators^6,16,53^ and aversive for vertebrate predators, specifically for lizards^54^, geckos^55^, house shrews (Fuchsová *et al*. in prep.) and passerine birds (Exnerová *et al*. in prep.). These results suggest that there might be a possible trade-off between the two lines of chemical defence, with the bugs raised on cardenolide-producing host plant investing less to the defensive components of their scent-gland secretion. On the other hand, it seems that the metathoracic scent glands, which are somewhat reduced in Lygaeinae and are regarded as having mostly pheromonal function^6^, might represent a more flexible system, which interacts with the alternative defence mechanism based on sequestration of host-plant chemicals.

## Conclusions

Composition of the defensive secretion of males and females of the milkweed bug *Lygaeus equestris* was analysed using 1D GC-MS and GC×GC-MS techniques. The bugs were raised either on a toxic, cardenolide-producing host plant or on a control sunflower. The secretion was sampled by slightly compressing the bugs with the plunger of a syringe and subsequent SPME analysis. Separation in a 1D GC-MS system with a mid-polar Rtx-200 column and also with the low-polar SLB-5ms column was not sufficiently effective for some substances, which co-eluted. The use of GC×GC-MS with an orthogonal polarity columns enabled separation or at least identification of the formerly co-eluting substances, such as (*E*)-2-octen-1-ol, (*E*)-2-octenal, decanal, 3-octen-1-ol acetate, (*E,E*)-2,4-hexadienyl acetate, 2,6-dimethyl-7-octen-2-ol and 1-octanol, which play an important role in antipredatory defence or intraspecific communication. Dominant components in the secretion of bugs raised on sunflower were (*E*)-2-octenal, (*E*)-2-octen-1-ol, decanal and 3-octen-1-ol acetate. Chromatograms obtained for the defensive secretions of sunflower-raised males and females displayed practically the same pattern of the identified substances and differences were observed only in their relative contents. Substantially greater intersexual differences were observed in the bugs raised on *A. vernalis*. Several components were identified only in the secretion of males, particularly (*E,E*)-2,4-hexadienyl acetate and 2-phenylethyl acetate with potential pheromonal function.

The bugs raised on *A. vernalis* exhibited lesser willingness to release scent-gland secretion than the bugs from sunflower, and also the intensity of peaks obtained for the compounds detected in the secretion of *A. vernalis*-raised bugs was lower. Moreover, some of the compounds that were dominant in the secretion of sunflower-raised bugs were entirely absent (or only present in low amounts) in the secretion of bugs raised on *A. vernalis*. These included chemicals with antipredatory defensive or signalling function, e.g. (*E*)-2-hexenal, (*E*)-4-oxo-hex-2-enal, 2,4-octadienal. The results indicate a possible trade-off between the sequestration of defensive chemicals from host plants and their autogenous synthesis in metathoracic scent-glands.
